# Change in Antimicrobial Therapy Based on Bronchoalveolar Lavage Data Improves Outcomes in ICU Patients with Suspected Pneumonia

**DOI:** 10.1155/2023/6928319

**Published:** 2023-08-14

**Authors:** Bharti Chogtu, Vrinda Mariya Elenjickal, Dharma U. Shetty, Mahsheeba Asbin, Vasudeva Guddattu, Rahul Magazine

**Affiliations:** ^1^Department of Pharmacology, Kasturba Medical College, Manipal Academy of Higher Education, Manipal, Karnataka 576104, India; ^2^Department of Respiratory Medicine, Kasturba Medical College, Manipal Academy of Higher Education, Manipal, Karnataka, 576104, India; ^3^Department of Data Science, Prasanna School of Public Health, Manipal Academy of Higher Education, Manipal, Karnataka, 576104, India

## Abstract

Flexible bronchoscopy (FB) is often performed in critically ill patients with suspected pneumonia. It is assumed that there will be an association with improved outcomes when bronchoalveolar lavage (BAL) data lead to a change in antimicrobial therapy. *Methods.* This study included a retrospective cohort of intensive care unit (ICU) patients who underwent FB for a diagnosis of suspected pneumonia. The study compared the outcome of patients in whom antimicrobial modification was carried out based on BAL reports versus those in whom it was not carried out. Cases where the procedure could not be completed or had incomplete records were excluded. The FB reports were accessed from the register maintained in the Department of Respiratory Medicine. The demographic details, clinical symptoms, laboratory investigations, and microbiological and radiology reports were recorded. Data on the antmicrobial therapy that the patients received during treatment and the outcome of the treatment were obtained from the case records and noted in the data collection form. *Results.* Data from a total of 150 patients admitted to the ICU, who underwent FB, were analyzed. The outcomes in the group where antimicrobial modification based on bronchoalveolar lavage (BAL) fluid reports was carried out versus the no-change group were as follows: expired 23, improved 82, unchanged 8 versus expired 12, improved 18, and unchanged 7 (*p* = 0.018); total duration of ICU stay 13.12 ± 10.61 versus 19.43 ± 13.4 days (*p* = 0.012); and duration from FB to discharge from ICU 6.33 ± 3.76 days versus 8.46 ± 5.99 (*p* = 0.047). The median total duration of ICU stay and clinical outcomes were significantly better in the nonintubated patients in whom BAL-directed antimicrobial modification was implemented. Distribution of microorganisms based on BAL reports was as follows: *Acinetobacter baumanii* 45 (30%), *Klebsiella pneumoniae* 37 (24.66%), *Escherichia coli* 9 (6%), and *Pseudomonas aeruginosa* 9 (6%). *Conclusion.* A change in antimicrobial therapy based on BAL data was associated with improved outcomes. The commonest bacterial isolate in the BAL fluid was *Acinetobacter baumanii*.

## 1. Introduction

Flexible bronchoscopy (FB) is an important diagnostic and therapeutic procedure in the management of respiratory disorders. It has been proven to be a relatively safe procedure, not only in the outpatient setting but also in the ICU, with a major complication rate of 0.08–2% and mortality of 0.01% [1–5]. In mechanically ventilated patients, 24 hours after bronchoscopy, a mortality of 1.77% has been observed [6]. Done early, bronchoscopy can positively impact the clinical outcome of patients admitted in an ICU setting [7]. Usually, critically ill patients presenting with sepsis due to a focus in the lungs are started on empirical antibiotics, pending sputum culture, and sensitivity results. Many a time this empirical treatment does not produce a favourable clinical response. In addition, in cases where the response to antimicrobial therapy, which is based on the sputum culture and sensitivity, is suboptimal, an invasive diagnostic procedure such as FB can be considered to obtain bronchoalveolar lavage (BAL) fluid [8]. The BAL fluid can then be subjected to laboratory analysis, and the microbial isolates thus identified can help guide therapy and potentially improve outcome [9, 10]. On the other hand, the sputum cultures have shown a positivity rate of only 40–50% for common bacterial microbes, since the results vary depending on whether the patient has provided a proper sample or not [11].

With this background, the present retrospective study was conducted to probe the impact of BAL report-directed antimicrobial modification on clinical management and the outcome of suspected pneumonia patients admitted to the ICU. This study also evaluated the spectrum of organisms grown from the BAL fluid.

## 2. Materials and Methods

This retrospective study was carried out at a tertiary care teaching hospital of a university medical college in South India. Approval for conducting the study was obtained from the institutional ethics committee (IEC: 525/2020, dated 09 September 2020). For this descriptive study, the case records of 203 consecutive patients, who were admitted to the ICU between January 2017 and August 2020 and underwent FB, were accessed from the medical records department of the hospital. Cases included in the study were as follows: all the ICU patients who underwent FB for a diagnosis of suspected pneumonia. The diagnosis of pneumonia was made by the treating physician based on guidelines by the Infectious Diseases Society of America and the American Thoracic Society (new infiltrate on chest X-ray along with clinical evidence of its infectious origin, which encompasses fever, purulent sputum, and leucocytosis) [11, 12]. Cases excluded from the study were as follows: ICU patients where FB could not be completed or those cases where the records were incomplete.

In case more than one FB had been carried out, then only the first FB reports were accessed. The FB reports were accessed from the register maintained in the Department of Respiratory Medicine. The demographic details regarding age, gender, clinical features, laboratory investigations, and microbiological reports were recorded. Chest X-ray and latest chest computed tomography (before FB) reports were also recorded. FB was performed by experienced pulmonologists using a Pentax video bronchoscope with a 6.4 mm insertion tube and a 2.8 mm instrument channel. Five minutes prior to the start of the procedure, the fraction of inspired oxygen (FiO_2_) was increased to 1.0 as per the ICU protocol. Intensive care physicians decided the level of sedation based on the patients' requirements and clinical status. BAL was carried out with the help of FB, with sterile normal saline instilled into the involved bronchopulmonary segments. Using a syringe, the instilled saline was then suctioned and collected in sterile containers. Retrieved BAL fluid was sent for various microbiologic tests including an aerobic bacterial quantitative culture using 5% sheep blood agar, MacConkey's agar, and chocolate agar. The next day, the growth on the Petri dishes was observed, and bacterial isolates were identified according to the standard protocol. Bacterial drug sensitivity tests (Kirby–Bauer disk-diffusion method) were performed. The samples from patients with suspected tuberculosis were processed for the cartridge-based nucleic acid amplification test (CBNAAT) by Cepheid, utilizing the technique of polymerase chain reaction (PCR). The mycobacterial culture was carried out using Mycobacteria Growth Indicator Tube (MGIT). Data on the antibiotic therapy that the patients received during treatment and the outcome of the patients were obtained from the case records and noted in the data collection form.

### 2.1. Statistical Methods

All the collected data were entered into an Excel sheet. Statistical Package for Social Sciences (SPSS) version 20.0 was used for the data analysis. Descriptive statistics were employed, and numbers and percentages were used to express the data. The chi-square test and the independent *t*-test were used for analyzing statistical significance, and a *p* value < 0.05 was considered significant.

## 3. Results

Postscreening, a total of 150 patients admitted to the ICU, who underwent FB, were included in this retrospective study. The demographic characteristics of the patients are summarized in Table 1.

### 3.1. The FB Findings

The FB findings were as follows: purulent secretions in 88 (58.7%), erythematous mucosa in six (4%) patients, fragile mucosa that bleeds on touch in seven (4.7%), mucus plug in three (2%), intraluminal growth in three (2%), extrinsic compression in four (2.7%), architectural distortion in one (0.7%), and no abnormality in 38 (25.3%). Four patients (2.7%) out of 150 underwent repeat FB.

### 3.2. Pre-FB Sputum/Endotracheal Tube Aspirate Culture

The following organisms grew in the culture: *Klebsiella pneumoniae* 13 (8.6%), *Pseudomonas aeuginosa* 5 (3.3%), methicillin-resistant *Staphylococcus Aureus* 4 (2.6%), *Acinetobacter baumanii* 3 (2%), *nocardia* 1 (0.66%), and sterile 124 (82.67%). Out of a total of 150 cases, the pre-FB blood culture showed the following distribution of organisms: *Acinetobacter baumanii* 4 (2.7%), *Klebsiella pneumonia* 2 (1.3%), *Candida parapsilosis* 2 (1.3%), *Enterococcus faecalis* 1 (0.7%), methicillin-resistant *Staphylococcus Aureus* 1 (0.7%), and sterile sample 140 (93.3%). The distribution of microorganisms based on BAL fluid reports is shown in Table 2.

The antimicrobial sensitivity pattern of the two most common BAL bacterial isolates is shown in the supplementary table. The antimicrobials prescribed prebronchoscopy and postbronchoscopy, respectively, were as follows: beta lactams (126, 68), macrolides (43, 10), aminoglycosides (8, 18), clindamycin (8, 11), linezolid (6, 5), doxycycline (4, 2), teicoplanin (4, 6), vancomycin (4, 3), quinolones (3, 5), metronidazole/tinidazole (3, 1), tigecycline (1, 15), colistin (0, 10), polymyxin (0, 13), cotrimoxazole (2, 10), and fosfomycin (0, 1). Among the 113 patients who had antimicrobial modification, 90 had complete replacement of their prebronchoscopy antimicrobials, while 23 had only the addition of new antimicrobials while continuing the usage of some or all of the prebronchoscopy antimicrobials. Of all the study subjects, 86 (57.30%) did not require invasive mechanical ventilation but were in ICU due to worsening clinical condition and the need for noninvasive ventilation. 62.9% (22) of patients who needed mechanical ventilation expired, compared to only 37.1% (13) who did not require mechanical ventilation (*p*=0.016). Of the 13 patients who expired in the nonintubated group, none was ever intubated before FB, but nine were intubated later before they expired.

Outcomes at discharge based on post-FB diagnosis were as follows: community-acquired pneumonia 17 expired, 57 improved, and 7 remained unchanged (this was as per the physician's assessment marked in the case records); hospital-acquired pneumonia 3 expired, 16 improved, and 0 remained unchanged; ventilator-associated pneumonia 8 expired, 9 improved, and 3 remained unchanged; aspiration pneumonia 1 expired, 2 improved, and 2 remained unchanged; and undiagnosed consolidation 6 expired, 16 improved, and 3 remained unchanged (*p*=0.10). Duration-related outcome measures and clinical outcomes of patients based on the implementation of BAL report-directed antimicrobial modification are shown in Table 3.

It was found that 67% of the patients, who underwent bronchoscopy, improved at the time of discharge, 10% had unchanged clinical outcomes, and 23% of the patients expired. Also, 47 (30.67%) patients had some comorbidity. Twelve (25.5%) of the patients with comorbidities expired, while 23 (22.3%) of the patients without comorbidities also expired (*p* value = 0.66).

## 4. Discussion

Diagnosing the aetiology of suspected pneumonia in ICU patients is likely to help in the choice of antimicrobials that are used for treatment. In a study from Spain, conducted on mechanically ventilated patients of community-acquired and ventilator-associated pneumonia, the investigators found that BAL fluid was useful in diagnosing the aetiology of ventilator-associated pneumonia [13]. A retrospective study, conducted in China, observed a reduction in mortality among patients with ventilator-associated pneumonia who were subjected to bronchoscopy, compared to those who were not [14]. Similarly, in our study, improved clinical outcomes in the antimicrobial-modified group suggest that therapeutic decisions based on the BAL fluid culture and sensitivity can potentially improve the management of these critically ill patients. Even the median total duration of hospital stay and the median total duration of ICU stay were significantly shorter in this group. In a study conducted on patients admitted to ICU with aspiration pneumonitis, the investigators observed a significant reduction (60.5 vs. 81.6%) in the rate of development of aspiration pneumonia in patients undergoing early bronchoscopy. A trend toward reduction in mortality and duration of mechanical ventilation was also observed, though not statistically significant [7]. In our study, nonintubated patients had significantly better clinical outcomes and a shorter median total duration of ICU stay than the intubated patients Table 4. This implies that antimicrobial modification can have a positive impact on cases that had less disease severity. The mortality risk increases in ventilator-associated pneumonia patients if antibiotic administration is delayed [15]. Hence, to achieve better outcomes, delay in initiating antibiotics and use of inadequate, empirical antibiotics should be avoided. Given the usefulness of BAL in obtaining samples for the culture and sensitivity, BAL-directed antimicrobial modification has the potential to positively impact mortality particularly among nonintubated ICU patients, as is suggested by results obtained in our study.

The spectrum of BAL fluid microbial isolates obtained from patients admitted to ICU varies across studies. In our study, *Acinetobacter baumanii* was the commonest bacterial isolate found, and similar results were obtained from studies conducted in ICU settings in Brazil, Mexico, India, and Iran, where one-third or more of the isolates were of *Acinetobacter baumanii* [3, 8, 16–18]. Contrary to this in a study from an Egyptian teaching hospital, the commonest isolate in the ICU setting was *Pseudomonas aeruginosa*, followed by *Acinetobacter* [2]. In a retrospective study from the United States, the most common organism in patients suffering from nosocomial and ventilator-associated pneumonia in the ICU setting was *Pseudomonas aeruginosa* (40%), followed by *Stenotrophomonas maltophilia* (34%) and *Acinetobacter baumannii* (32%) [19]. In studies from India and Nepal, *Pseudomonas aeruginosa* was the commonest organism isolated from BAL fluid of patients, but this was in a non-ICU setting [20, 21]. As is evident from these studies, the spectrum of organisms varied, depending on the clinical diagnosis (community-acquired pneumonia or nosocomial pneumonia or ventilator-associated pneumonia), treatment setting (ICU or non-ICU), and the geographic location of the study.

In patients admitted to ICU, BAL has been reported to influence the choice of therapy in 29% to 54.8% of cases [6, 8, 22]. In our study, BAL fluid-directed antimicrobial modification (introduction of at least one new antimicrobial, with or without stopping current antimicrobials) was possible in three-fourths of the patients (antimicrobial modified group), while in one-fourth, no new antimicrobials were introduced as the BAL fluid culture was sterile (no-change group). The variance between the studies could be due to the differences in patient characteristics such as indications for FB, which in turn would influence the need for BAL fluid sampling. However, it is evident from all these studies that BAL fluid reports influenced the line of treatment in a substantial percentage of patients in the ICU.

Given the evolving antimicrobial resistance to antibiotics, it seems logical to obtain BAL fluid or any other relevant sample promptly and subject it to the bacterial culture and sensitivity. BAL has been shown to have a better sensitivity and specificity than the Clinical Pulmonary Infection Score, which is based on readily available clinical, radiographic, and microbiological data [23]. Since there are studies in favour as well as against the use of BAL in ICU patients, the prudent approach may be to choose it as a diagnostic modality on a case-to-case basis, depending on the clinical setting and locally available resources [7, 8, 13, 22, 24, 25]. In such a situation, the clinician's personal experience and preference also tend to play an important role in selecting a diagnostic modality. The effort should be to make decisions based on the available evidence in the literature.

## 5. Limitations

Being a retrospective study, some cases could not be included due to inadequacy of data. This led to 25% of screened subjects being excluded from the study. There was higher percentage of subjects above 50 years of age and with “other” comorbidities in the no-change group at baseline. In the latter, the difference was statistically significant. A single-centre, descriptive design of the study makes the results difficult to generalize. In addition, FB was performed only by pulmonologists, whereas in many centres, intensivists also perform this procedure. The data on viral aetiology of pneumonia were not captured; thus, the complete overview of the microbial spectrum could not be obtained. Also, clinical data regarding patients' severity were not recorded. Advanced hypothesis testing such as logistic regression analysis was not possible in this study, considering the small sample size in the group where antibiotics were not modified.

## 6. Conclusion

A change in antimicrobial therapy based on BAL data was associated with improved mortality and decreased ICU length of stay. Nonintubated patients had significantly better clinical outcomes and a shorter median total duration of ICU stay than the intubated patients. The commonest bacterial isolate in the BAL fluid obtained from ICU patients was *Acinetobacter baumanii*.

## Figures and Tables

**Table 1:** Demographic characteristics of patients (*n* = 150).

Demographic data	Overall (*n* = 150)	Antimicrobial modified group (*n* = 113)	No-change group (*n* = 37)	*p* value
*Age group*				
<20	5 (3.3)	4 (3.54)	1 (2.70)	0.15
20–50	33 (22)	29 (25.66)	4 (10.81)	
>50	112 (74.7)	80 (70.80)	32 (86.49)	

*Gender*				
Male	100 (66.67)	74 (65.49)	26 (70.27)	0.59
Female	50 (33.33)	39 (34.51)	11 (29.73)	
Smoking	52 (34.7)	39 (34.51)	13 (35.14)	0.95

*Comorbidities*				
Diabetes mellitus	44 (29.3)	33 (29.2)	11 (29.73)	0.95
Hypertension	46 (30.7)	33 (29.20)	13 (35.14)	0.41
Chronic kidney disease	13 (8.7)	7 (6.19)	6 (16.22)	0.061
Ischemic heart disease	5 (3.3)	4 (3.54)	1 (2.70)	0.81
Others	11 (7.3)	6 (5.31)	5 (13.51)	0.01

Prebronchoscopy diagnosis *n* (%)				
Community-acquired pneumonia	81 (54.0)	63 (55.75)	18 (48.65)	0.49
Ventilator-associated pneumonia	20 (13.3)	16 (14.16)	4 (10.81)	
Hospital-acquired pneumonia	19 (12.7)	12 (10.62)	7 (18.92)	
Lung collapse (with suspected pneumonia)	15 (10.0)	10 (8.85)	5 (13.51)	
Aspiration pneumonia	5 (3.3)	3 (2.65)	2 (5.41)	
Others (with suspected pneumonia)	10 (6.7)	9 (7.96)	1 (2.70)	

*Route of bronchoscope insertion n (%)*				
Trans endotracheal tube	63(42)	47 (41.59)	16 (43.24)	0.66
Trans nasal	77 (51.3)	57 (50.44)	20 (54.05)	
Trans oral	4 (2.7)	4 (3.54)	0	
Trans tracheostomy	6 (4)	5 (4.4)	1 (2.7)	
Invasive mechanical ventilation	64 (42.7)	48 (42.5)	16 (43.2)	0.94

^*∗*^Chi-square test; a *p* value is for the difference between the two groups, i.e., the antimicrobial modified group and the no-change group. Seven patients (4.7%) were human immune deficiency virus positive (HIV-positive). The most common symptom at presentation was cough in 107 patients (71.33%) followed by breathlessness in 97 patients (64.66%). Other symptoms included sputum production (*n* = 72 (48%)), fever (*n* = 71 (47.33%)), and haemoptysis (*n* = 4 (2.67%)).

**Table 2:** Distribution of microorganisms based on BAL reports (postbronchoscopy).

Microorganisms	*n* (%)
*Acinetobacter baumanii*	45 (30)
*Klebsiella pneumoniae*	37 (24.66)
*Escherichia coli*	9 (6)
*Pseudomonas aeruginosa*	9 (6)
MRSA	2 (1.33)
*Burkholderia pseudomallei*	2 (1.33)
*Staphylococcus aureus*	1 (0.66)
*Streptococcus pneumoniae*	1 (0.66)
*Mycobacterium tuberculosis*	7 (4.67)
Sterile	37 (24.67)
Total	150 (100)

Post-FB diagnosis was as follows: community-acquired pneumonia 81 (54%), ventilator-associated pneumonia 20 (13.3%), hospital-acquired pneumonia 19 (12.7%), aspiration pneumonia 5 (3.3%), and undiagnosed consolidation 25 (16.7%).

**Table 3:** Outcome measures of patients based on implementation of BAL report-directed antimicrobial modification.

	*Antimicrobial modified*		*p* value
	Yes (*n* = 113)	No (*n* = 37)	
*Duration-related outcomes*			
Total duration of hospital stay (median, (IQR))	16 (12–25)	20 (15–37)	0.042^*∗*^
Total duration of ICU stay (median, (IQR))	10 (7–15)	15 (10–30)	0.003^*∗*^
Duration from admission to FB (median, (IQR))	6 (3–10)	7 (5–11)	0.502^*∗*^
Duration from FB to discharge from ICU (median, (IQR))	6(3–10)	7(5–11)	0.111^*∗*^
Duration from FB to discharge from hospital (median, (IQR))	6 (4–10)	8 (5–13)	0.064^*∗*^

*Clinical outcomes*			
Expired, *n* (%)	23 (20.4)	12 (32.4)	
Improved, *n* (%)	82 (72.5)	18 (48.7)	0.018^#^
Unchanged, *n* (%)	8 (7.1)	7 (18.9)	

^*∗*^Mann–Whitney *U* test; ^#^chi-square test; IQR: interquartile range.

**Table 4:** Outcome measures of intubated versus nonintubated patients based on BAL report-directed antimicrobial modification.

	Not intubated		*p* value	Intubated		*p* value
	Antimicrobial modified			Antimicrobial		
				Modified		
	Yes (*N* = 65)	No (*N* = 21)		Yes (*N* = 48)	NO (*N* = 16)	
*Duration-related outcomes * ^*∗*^						
Total duration of hospital stay (median, (IQR))	17 (13–25)	21 (15–37)	0.200	15 (11.5–24)	20 (14.5–35)	0.123^*∗*^
Total duration of ICU stay (median, (IQR))	10 (7–16)	15 (12–35)	0.001	10 (8–15)	11 (6.5–22.5)	0.641^*∗*^
Duration from admission to FB (median, (IQR))	7 (3–11)	8 (5–11)	0.860	5 (3–8)	5.5 (4.5–11.5)	0.483^*∗*^
Duration from FB to discharge from ICU (median, (IQR))	6 (4–10)	10 (4–12)	0.201	5 (3–9)	6 (5–10)	0.291^*∗*^
Duration from FB to discharge from hospital (median, (IQR))	6 (4–11)	9 (5–15)	0.080	6 (3.5–9.5)	6 (5–12)	0.435^*∗*^

*Clinical outcomes * ^#^						
Expired, *n* (%)	8 (12.3)	5 (23.8)	0.03	15 (31.2)	7 (43.8)	0.33^#^
Improved, *n* (%)	54 (83.1)	12 (57.2)		28 (58.3)	6 (37.5)	
Unchanged, *n* (%)	3 (4.6)	4 (19)		5 (10.5)	3 (18.7)	

^*∗*^Mann–Whitney *U* test; ^#^chi-square test. IQR: interquartile range.

## Data Availability

The data used to support the study are available from the corresponding author upon request.

## Supplementary Materials

Supplementary Materials Supplementary Table: antimicrobial sensitivity pattern of two most common BAL bacterial isolates.
