# Supplementary material for: Change in Antimicrobial Therapy Based on Bronchoalveolar Lavage Data Improves Outcomes in ICU Patients with Suspected Pneumonia
Source: Crit Care Res Pract. 2023 Aug 14;2023:6928319. doi: 10.1155/2023/6928319 (PMC10442184; doi:10.1155/2023/6928319)
Supplement: Supplementary Materials — Supplementary Table: antimicrobial sensitivity pattern of two most common BAL bacterial isolates. [file 6928319.f1.docx]

**Supplementary Table: Antimicrobial Sensitivity Pattern of Two Most Common BAL Bacterial Isolates**

|  | **SENSITIVITY OF ISOLATES***  **n/N** | |
| --- | --- | --- |
| **Antimicrobial Agent** | ***Acinetobacter baumanii*** | ***Klebsiella pneumonia*** |
| Amikacin | 5/36 (13.9) | 20/37 (54) |
| Gentamicin | 6/40 (15) | 23/37 (62.2) |
| Cefoperazone Sulbactam | 3/42 (7.1) | 11/37 (29.7) |
| Tigecycline | 26/39 (66.7) | 11/24 (45.8) |
| Amoxicillin clavulanic acid | 0/42 (0) | 7/36 (19.4) |
| Ciprofloxacin/ofloxacin | 1/42 (2.4) | 4/37 (10.8) |
| Cefotaxime/ ceftriaxone | 0/43 (0) | 3/37 (8.1) |
| Cefepirome/cefepime | 0/41 (0) | 8/37 (21.6) |
| Trimethoprim sulphmethoxazole | 2/41 (4.9) | 4/37 (10.8) |
| Meropenem | 2/ 40 (5) | 15/37 (40.5) |
| Imipenem | 2/42 (4.8) | 16/37 (43.2) |
| Piperacillin tazobactam | 1/42 (2.4) | 9/37 (24.3) |

* Number of isolates sensitive to a particular antimicrobial divided by total number of isolates tested for that antimicrobial sensitivity × 100
